# Supplementary material for: Genomic insights into the expansion of meropenem-resistant GPSC1-CC320 Streptococcus pneumoniae serotype 19A isolates from children under 5 years of age with invasive infections, 2018–2024
Source: PLoS One. 2025 Dec 30;20(12):e0325870. doi: 10.1371/journal.pone.0325870 (PMC12752945; doi:10.1371/journal.pone.0325870)
Supplement: Attachment — Submitted filename: reviewer_notes.docx [file pone.0325870.s001.docx]

This study highlights a notable reemergence of serotypes 19A prevalence and disease post-PCV introduction, provides explanation of resistance mechanisms, and potential reasoning behind the increase in 19A prevalence. All of which highlights the importance to monitor this specific serotype in the South Korean children population. Overall, the manuscript reads well and is clearly written. There is originality and significance to the dataset, however, additional issues (major and minor) need to be addressed.

Major issues

1. The study claims “19A is driven by stable, high-risk MDR clones rather than vaccine failure alone.” However, the evidence for this is not very convincing e.g. besides the evidence that these 19A isolates resembles those that are found in other parts of the world – is it difficult to exclude that these 19A disease isolates are not a result of breakthrough infections.
2. Line 150-152: “markedly increased in recent years…rate increasing significantly” - was there stat significant difference in the increase? If so, there’s no mention of what stats were used to test significance. Are these results supported by figure 1, if so, cite figure 1.
3. Line 192: Generally, the discussion is lacking e.g. it is advisable to include a limitations section to the study.

Minor issues

4. Line 54: write out acronym “PSERENADE”
5. Line 63: what are examples of region-specific adaptations?
6. Line 66: why report on uptake rate and not coverage rate?
7. Line 70: sometimes just “Korea” is written. Try to be consistent and use “South Korea” and ” South Korean” etc throughout the whole manuscript.
8. Line 84: of the 104 isolates, what were the distribution of the isolates over the years?
9. Line 89: “species” – clarify what you mean by species e.g. presence of S pneumo
10. Line 91: “after confirmation” – clarify what you mean by this e.g. confirmation of presence of S pneumo
11. Line 107: how many samples were CSF and nonCSF?
12. Line 135: write out acronym for “KDCA” and “IRB”
13. Line 155: “tested” – change to “were tested”
14. Line 156: “All the isolates...” – change to “All 11 isolates...”
15. Line 164-166: are you implying that the presence of that specific PBP type combination explains the intermediate or resistance to those antibiotics? If so, is there evidence/reference that supports this?
16. Line 178: “The GPSC 1 isolates” – clarify that the study isolates here
17. Line 179: “3, 6C, 14, 19A, 19F and 23F serotypes” – change to “serotypes 3, 6C, 14, 19A, 19F and 23F”
18. Line 200: “No. 19A” – what do you mean? The number of 19A isolates?
