# Supplementary material for: Genomic insights into the expansion of meropenem-resistant GPSC1-CC320 Streptococcus pneumoniae serotype 19A isolates from children under 5 years of age with invasive infections, 2018–2024
Source: PLoS One. 2025 Dec 30;20(12):e0325870. doi: 10.1371/journal.pone.0325870 (PMC12752945; doi:10.1371/journal.pone.0325870)
Supplement: Attachment — Submitted filename: responses to reviewer.docx [file pone.0325870.s002.docx]

Responses to reviewer’s comments

**[Manuscript ID: PONE-D-25-27201]**

**Genomic and Epidemiological Dynamics of Meropenem-Resistant GPSC1-CC320**

**Streptococcus pneumoniae Serotype 19A Isolates from Children Under 5 Years of Age**

**with Invasive Infections, 2018–2024**

**● Reviewer #1**

1. The study claims “19A is driven by stable, high-risk MDR clones rather than vaccine failure alone.” However, the evidence for this is not very convincing e.g. besides the evidence that these 19A isolates resembles those that are found in other parts of the world – is it difficult to exclude that these 19A disease isolates are not a result of breakthrough infections.

- Response: We sincerely appreciate the reviewer’s insightful comment. We agree that, due to the lack of individual vaccination history data in our dataset, it is not possible to exclude the potential contribution of vaccine breakthrough infections to the recent increase in serotype 19A invasive pneumococcal disease (IPD). To address this concern, we have revised the Discussion section to clarify our interpretation and provide a balanced perspective on the role of clonal expansion versus vaccine breakthrough infections:

Revised text (Discussion, Page 11, Lines 218–223):

“Furthermore, our phylogenetic analysis demonstrated that South Korean 19A isolates formed four distinct subclades interspersed within international ST320/ST6400 lineages, sharing highly conserved PBP alleles and MDR gene profiles. These findings suggest that the recent resurgence of 19A may be largely associated with the clonal expansion of stable, high-risk MDR lineages. However, given the lack of vaccination history data, we cannot fully exclude the possibility that a proportion of these cases may represent vaccine breakthrough infections.”

Additionally, we have reinforced this point in the Limitations section (Page 13, Lines 260–263) by noting that the absence of vaccination history data limited our ability to fully evaluate the contribution of vaccine breakthrough infections. We also emphasize that future studies incorporating detailed vaccination records will be essential to clarify the relative contributions of clonal expansion and vaccine-induced immunity in the re-emergence of serotype 19A.

2. Line 150-152: “markedly increased in recent years…rate increasing significantly” - was there stat significant difference in the increase? If so, there’s no mention of what stats were used to test significance. Are these results supported by figure 1, if so, cite figure 1.

- Response: We appreciate the reviewer’s valuable comment. As noted, the original manuscript did not sufficiently specify the statistical methods used to evaluate the observed differences. To address this, we have clarified that the statistical analyses were performed using Fisher’s exact test, provided the corresponding p-values, and cited Figure 1 to indicate that the results are visually supported. We have also updated the Figure 1 caption to include the statistical significance.

Revised text (Results, Page 8, Lines 157–162):

“The proportion of PCV13 serotypes has markedly increased in recent years, increasing from 9.1% (2/22) in 2018 to 27.8% (5/18) in 2023 and reaching 31.6% (6/19) in 2024. Notably, serotype 19A was the most predominant PCV13 serotype, with its detection rate increasing from 9.1% (2/22) in (2018) and 14.3% (1/7) in (2020) to 16.7% (3/18) in (2023) and 26.3% (5/19) in (2024). Furthermore, a comparison between the two periods (2018–2022 vs. 2023–2024) demonstrated significant increases in both the overall proportion of PCV13 serotypes (p = 0.0023) and serotype 19A specifically (p = 0.0154) (Fig 1).”

Revised Figure 1 caption:

“Fig 1. Distribution of PCV13 and non-PCV13 serotypes among invasive *S. pneumoniae* isolates from Korean children under 5 years of age, 2018–2024.

(A) Annual distribution of serotype 19A among 104 *S. pneumoniae* isolates from children <5 years of age with IPD in South Korea, 2018–2024.

(B) Comparison of PCV13 versus non-PCV13 isolates (left) and serotype 19A versus non-19A isolates (right) between the periods 2018–2022 and 2023–2024. Statistical significance was determined using Fisher’s exact test (left: p = 0.0023; right: p = 0.0154)”

3. Line 192: Generally, the discussion is lacking e.g. it is advisable to include a limitations section to the study.

- Response: We thank the reviewer for this important suggestion. In response, we have added a dedicated Limitations section to the manuscript to clearly outline the key constraints of our study:

New Limitations Section (Page 12, Lines 256–263):

“This study has several limitations. First, pneumococcal serotype analysis was recommended but not mandated under the Infectious Diseases Surveillance Systems, and thus the data may not fully represent the entire population. Consequently, these findings should be interpreted with caution, as this limitation may affect the interpretation of surveillance trends and subsequent analyses.

Second, due to the lack of individual vaccination history data, we were unable to fully evaluate the contribution of vaccine breakthrough infections to the resurgence of serotype 19A. Future studies incorporating detailed vaccination records will be essential to better clarify the relative roles of clonal expansion and vaccine-induced immunity in the re-emergence of serotype 19A.”

4. Line 54: write out acronym “PSERENADE”

- Response: Thank you for pointing this out. We have written out the full name of PSERENADE upon its first mention in the Introduction (Page 3, Line 54-55).

5. Line 63: what are examples of region-specific adaptations?

- Response: We appreciate the reviewer’s insightful suggestion. We have clarified the text by providing specific examples of region-specific adaptations, including variations in β-lactam resistance-associated pbp alleles and recombination events leading to capsular switching [8,10], and added Reference [10] to support this statement (Page 4, Lines 64–65).

10.	Puzia W, Gawor J, Gromadka R, Żuchniewicz K, Wróbel-Pawelczyk I, et al. Highly resistant serotype 19A Streptococcus pneumoniae of the GPSC1/CC320 clone from invasive infections in Poland prior to antipneumococcal vaccination of children. Infect Dis Ther. 2023;12:2017–37. https://doi.org/10.1007/s40121-023-00842-w

6. Line 66: why report on uptake rate and not coverage rate?

- Response: We are grateful for the reviewer’s comment. The value of 88.6% was cited directly from Reference [11], which reports that “88.6% of infants are vaccinated with PCV13.” To maintain consistency and accuracy, we used the term “uptake rate” in the manuscript when referring to this data (Page 4, Lines 68).

7. Line 70: sometimes just “Korea” is written. Try to be consistent and use “South Korea” and ” South Korean” etc throughout the whole manuscript.

- Response: Thank you for the suggestion. We have thoroughly reviewed the manuscript and revised all instances to consistently use “South Korea

8. Line 84: of the 104 isolates, what were the distribution of the isolates over the years?

- Response: We appreciate the reviewer’s helpful suggestion. We have added the annual distribution of the 104 isolates in the Methods section for clarity: 2018 (22), 2019 (10), 2020 (7), 2021 (13), 2022 (15), 2023 (18), and 2024 (19) (Page 5, Lines 87–89).

9. Line 89: “species” – clarify what you mean by species e.g. presence of S pneumo

- Response: We have clarified that “species” refers to the confirmation of the presence of *S. pneumoniae* (Page 5, Line 92).

10. Line 91: “after confirmation” – clarify what you mean by this e.g. confirmation of presence of S pneumo

- Response: We appreciate the reviewer’s comment. We have revised the text by replacing “After confirmation” with “After confirming the identification of *S. pneumoniae*” to clarify that the confirmation refers specifically to the identification of *S. pneumoniae* (Page 5, Line 94).

11. Line 107: how many samples were CSF and nonCSF?

- Response: We thank the reviewer for this helpful comment. We have added the following information to the Methods section for clarification:

“Among the 11 serotype 19A isolates, one isolate was recovered from cerebrospinal fluid (CSF), and the remaining ten were from non-CSF sources.” (Page 6, Lines 111–112).

12. Line 135: write out acronym for “KDCA” and “IRB”

- Response: We thank the reviewer for this helpful comment. The full name of KDCA (Korea Disease Control and Prevention Agency) was already written out at its first mention in the Methods section. For IRB, we have added its full name (Institutional Review Board) at its first occurrence, as suggested. (Page 7, Lines 143)

13. Line 155: “tested” – change to “were tested”

- Response: Corrected as suggested (Page 8, Line 164).

14. Line 156: “All the isolates...” – change to “All 11 isolates...”

- Response: Revised as suggested (Page 8, Line 165).

15. Line 164-166: are you implying that the presence of that specific PBP type combination explains the intermediate or resistance to those antibiotics? If so, is there evidence/reference that supports this?

- Response: We thank the reviewer for this important point. We have clarified that the observed PBP type combinations are associated with intermediate resistance and non-susceptibility, and added Reference [15] to support this statement (Page 9, Line 175).

15.	Varghese R, Basu S, Neeravi A, et al. Emergence of meropenem resistance among cefotaxime non-susceptible Streptococcus pneumoniae: evidence and challenges. Front Microbiol. 2022;12:810414. https://doi.org/10.3389/fmicb.2021.810414

16. Line 178: “The GPSC 1 isolates” – clarify that the study isolates here

- Response: We thank the reviewer for the helpful suggestion. To clarify that the isolates refer specifically to those identified in this study, we have revised the text to:

“The GPSC1 isolates identified in this study” (Page 9, Line 187).

17. Line 179: “3, 6C, 14, 19A, 19F and 23F serotypes” – change to “serotypes 3, 6C, 14, 19A, 19F and 23F”

- Response: Revised as suggested (Page 10, Line 188).

18. Line 200: “No. 19A” – what do you mean? The number of 19A isolates?

- Response: We thank the reviewer for this helpful comment. To improve clarity, we have revised the sentence to:

“Notably, serotype 19A was not detected.” (Page 10, Line 209).

This concludes our response to the reviewer’s comments. We believe they have helped us improve our work considerably. We hope that they now find our manuscript acceptable for publication.
