# Supplementary material for: Genomic insights into the expansion of meropenem-resistant GPSC1-CC320 Streptococcus pneumoniae serotype 19A isolates from children under 5 years of age with invasive infections, 2018–2024
Source: PLoS One. 2025 Dec 30;20(12):e0325870. doi: 10.1371/journal.pone.0325870 (PMC12752945; doi:10.1371/journal.pone.0325870)
Supplement: Attachment — Submitted filename: (Plos one) Responses to reviewers_2.docx [file pone.0325870.s003.docx]

Responses to reviewer’s comments

**[Manuscript ID: PONE-D-25-27201]**

**Genomic Insights into the Expansion of Meropenem-Resistant GPSC1-CC320 Streptococcus pneumoniae Serotype 19A Isolates from Children Under 5 Years of Age with Invasive Infections, 2018–2024**

**● Reviewer #2**

1. In the abstract, the authors state the “Our findings suggest that the resurgence of 19A is driven by stable, high-risk MDR clones rather than vaccine failure alone”. The data provided by the authors did not allow them to suggest that vaccine failure happened or not. So, please remove “rather than vaccine failure alone”.

- Response: Thank you for this helpful comment. We agree that our data do not support any conclusion regarding vaccine failure. To avoid suggesting an interpretation that cannot be substantiated by our dataset, we removed the phrase “rather than vaccine failure alone” from the abstract. (Abstract, Page 2, Line 36)

2. Vaccine uptake and vaccine coverage may not have the same meaning. In the Introduction (line 68), the statement “… with PCV13 achieving an uptake rate of 88.6%” is not very clear. Is it the proportion of vaccinated children who took PCV13? If so, it does not reflect vaccine coverage. The authors need to show how the vaccine coverage was overtime, since the data they referenced are from 10 years ago. If no newer information about vaccine coverage, especially with PCV13, is available, the authors need to make it clear.

- Response: Thank you for raising this important point. We agree that the previously cited PCV13 uptake value was outdated and that the terminology used lacked clarity. To address this, we removed the outdated uptake figure and revised the Introduction to incorporate the most recent national immunization coverage data. “*According to the latest national report, PCV coverage among children in South Korea reached 97% in 2023*” and the corresponding reference has been updated accordingly. (Introduction, Page 4, Lines 68–69)

3. In the objective, the authors state that they will assess genomic and epidemiologic factors contributing to the re-emergence of serotype 19A. However, I did not find any epidemiological aspects discussed in this manuscript. Nothing related to the population was compared. I recommend removing the term “epidemiologic” from here and the abstract.

- Response: Thank you for this helpful observation. Our study does not include population-level epidemiological comparisons, and the use of the term “epidemiologic” could therefore be misleading. We carefully reviewed the manuscript and removed the term from all relevant sections, including the Title, Introduction, Method, and Discussion (Title; Introduction, Page 4, Line 77 and 81; Methods, Page 7, Line 140; Discussion, Page 10, Line 207)

4. Methods (lines112-113): “For isolates obtained from cerebrospinal fluid (CSF)…” Only one isolate was recovered from CSF. So, rephrase it. “For the single isolate recovered…”

- Response: Thank you for pointing this out. We revised the sentence to accurately indicate that only one isolate was obtained from CSF. (Methods, Page 6, Line 114)

5. Discussion (first paragraph): “These isolates, belonging to the GPSC1‒CC320 clonal complex, were analyzed to elucidate their resistance mechanisms and the epidemiological context of their re-emergence in a population with high PCV13 coverage”. Again, no classical epidemiological data were available in the study. Epidemiological data refer to the characteristics of the population from which the isolates were obtained. Vaccination history of the children was not available, for instance. So, this term should be removed.

- Response: We appreciate your clarification on this point. As our study does not include population-level epidemiological data, we removed the term “*epidemiological context*” from the Discussion and revised the sentence to focus solely on genomic and resistance-related findings. (Discussion, Page 10, Lines 207)

6. Also, I did not see any updated information on PCV13 coverage in the study. To keep this statement at the end of the first paragraph of the discussion, the authors need to include in the introduction session the vaccine coverage over the years of study or at least the most recent data available. At least, the PCV13 coverage during the study period needs to be stated in the introduction.

- Response: Your comment appropriately underscores the need updated vaccine coverage information. In response, we revised the Introduction to include the most recent national data indicating that PCV coverage among children in South Korea reached 97% in 2023, and updated the corresponding reference (Introduction, Page 4, Lines 68–69).

7. Limitations: change “several” to “some”. Also, in general, a paragraph with limitations comes before the final paragraph (conclusions).

- Response: We acknowledge your guidance regarding the structure of the Discussion section. Following your recommendation, we replaced “*several*” with “*some*” and repositioned the Limitations paragraph to appear immediately before the final summary paragraph (Discussion, Page 12, Lines 251–258).

This concludes our response to the reviewer’s comments. We believe that the revisions have substantially strengthened the clarity and scientific rigor of the manuscript. We appreciate the reviewer’s thoughtful assessment and hope that the revised version will be found suitable for publication.
